# Desensitization of transient receptor potential vanilloid type-1 (TRPV1) channel as promising therapy of irritable bowel syndrome: characterization of the action of palvanil in the mouse gastrointestinal tract

**DOI:** 10.1007/s00210-020-01829-x

**Published:** 2020-01-30

**Authors:** Agata Szymaszkiewicz, Jakub Włodarczyk, Andrzej Wasilewski, Vincenzo Di Marzo, Martin Storr, Jakub Fichna, Marta Zielińska

**Affiliations:** 1 Department of Biochemistry, Faculty of Medicine, Medical University of Lodz, Mazowiecka 6/8, 92-215 Lodz, Poland; 2 Endocannabinoid Research Group, Istituto di Chimica Biomolecolare, Consiglio Nazionale delle Ricerche, Pozzuoli, Italy; 3 Canada Excellence Research Chair on the Microbiome-Endocannabinoidome Axis in Metabolic Health, Université Laval, Quebec City, Canada; 4 Walter Brendel Center of Experimental Medicine, Ludwig Maximilians University, Munich, Germany; 5 Center of Endoscopy, Starnberg, Germany

**Keywords:** Capsaicin, Desensitization, Irritable bowel syndrome, Pain, Palvanil, TRPV

## Abstract

TRPV1 are involved in the control of the gastrointestinal (GI) functions and pain sensation. Their activation induces pain but it is followed by desensitization, which in turn causes analgesia. The studies from the last two decades indicate that TRPV1 are involved in visceral hypersensitivity in the GI tract and pathogenesis of irritable bowel syndrome (IBS). Therefore, the aim of this study is to assess the action of fast desensitizing agonist of TRPV1, palvanil (N-palmitoyl-vanillamine), in the murine GI tract and on nociception to evaluate its potential application in the therapy of IBS. The effect of palvanil on smooth muscle contractility was evaluated using organ baths. The impact of palvanil on intestinal secretion was assessed in Ussing chambers. In vivo, the action of palvanil (0.1–1 mg/kg) was assessed in whole GI transit, fecal pellet output, and colonic bead expulsion tests. The antinociceptive potency of palvanil was tested in the mustard oil-induced pain test. Palvanil inhibited colonic contractions (evoked by electrical field stimulation, EFS) and decreased the ion transport in the colon stimulated with forskolin. It did not affect secretion in experiments with veratridine. In vivo, palvanil prolonged whole GI transit at all doses tested. At the lower dose tested, it accelerated colonic motility during first 60 min following injection. By contrast, at the dose of 1 mg/kg, colonic motility was inhibited. Palvanil induced antinociceptive action at all tested doses in mustard oil-induced pain test. TRPV1 fast-desensitizing compounds, i.e., palvanil, may be promising agents in the therapy of IBS since it modulates intestinal motility and reduces visceral pain.

## Introduction

Irritable bowel syndrome (IBS) is a functional disease of the gastrointestinal (GI) tract, affecting nearly 15% of the world population (Longstreth et al. 2006). The course of IBS encompasses two essential issues: abnormalities in defecation pattern and abdominal pain. Undoubtedly, abdominal pain is related to the altered visceral sensation through peripheral or central sensitization processes. Peripheral mechanisms may be a result of the sensitization of nociceptive afferent nerve fibers or nociceptors through the altered expression of specific ion channels which are responsive to noxious stimuli (Holzer 2011).

TRPV 1 channels are known as “capsaicin receptors” since 1997, because of the piquancy effect evoked by capsaicin (a major ingredient of red pepper and TRPV1 ligand) (Julius et al. 1997). These channels are important in peripheral nociception, thermosensation, and inflammatory hyperalgesia (Julius 2013). The activation of TRPV1 leads to calcium ion entry into nociceptive sensory neurons leading to their depolarization and ultimately induces the release of algogenic compounds (calcitonin gene-related peptide (CGRP) and substance P) with activation of the ascending pathway engaged in pain transmission (Bölcskei et al. 2005). Notably, the increment of intracellular calcium ion concentration induces subsequent desensitization of TRPV1, which then becomes unresponsive to further stimulation with noxious triggers (heat or the action of algogenic neuromediators). As a consequence, the paradoxical antinociceptive action is observed (Touska et al. 2011).

The studies from the last two decades indicate that TRPV1 channels are involved in visceral hypersensitivity in the GI tract (Blackshaw et al. 2010) and pathogenesis of IBS. For example, Akbar et al. (2008) found that in IBS patient rectosigmoid biopsies, TRPV1-immunoreactive nerve fibers were 3.5-fold more prevalent than in healthy controls and their expression correlated with the severity of pain reported by patients. Balemans et al. (2017) investigated the underlying mechanism of hypersensitivity in post-infectious IBS (PI-IBS). In colonic biopsies from PI-IBS patients, submucosal neurons were significantly more sensitive to acute application of capsaicin (1 nM) as compared with those of the control group. However, opposite results were reported by van Wanrooij et al. (2013): the expression of TRPV1 did not correlate with the pain response to colorectal distension (CRD) after rectal installation of capsaicin. Noteworthy, Gonlachanvit et al. (2009) observed that patients with diarrhea-predominant IBS (IBS-D) were hypersensitive to spicy meal (containing chili or chili capsules) and that the ingestion of spicy food induced significantly more severe abdominal pain and burning sensation in IBS-D patients than in healthy controls (Gonlachanvit et al. 2009).

There were several attempts to apply capsaicin into clinics. For example, Bortolotti and Porta (2011) conducted a pilot study in which IBS patients received capsules containing red pepper (150 mg of red pepper/capsule) or placebo. It was observed that the abdominal pain score in the red pepper group decreased during the last 2 weeks of trial compared with baseline. In contrast, patients in the placebo group reported a slight improvement in pain scores within the first 4 weeks of the study, while this effect disappeared in the last 2 weeks. What is important: one third of the patients required reduction of capsaicin dose; another one fourth of the patients dropped the study because of abdominal pain aggravation. The effect of capsaicin in IBS patients was interpreted to be a result of TRPV1 sensitization (the primary, algogenic effect of capsaicin), followed by the desensitization phenomenon (the secondary effect—an improvement in pain scores). Notably, however, besides the lack of difference between the red pepper and placebo groups, patients experienced symptom improvement in comparison with baseline.

Therefore, in order to improve the potential application of TRPV1-related signaling in the therapy of IBS, capsaicin derivatives (i.e., olvanil (N-oleoyl-vanillamine), arvanil N-arachidonoyl-vanillamine, or palvanil (N-palmitoyl-vanillamine) deprived of pungent effect with ability to fast TRPV1 desensitization are considered a promising alternative to capsaicin (Luongo et al. 2012; De Petrocellis et al. 2011; Ursu et al. 2010).

Hereafter, this is the first available report on the action of fast desensitizing TRPV1 agonist on GI peristalsis, in which we assessed the action of palvanil on GI functions: motility and secretion using ex vivo and in vivo tests. Moreover, to evaluate the potential application of TRPV1 agonists in the therapy of IBS, we examined the antinociceptive potential of palvanil in a mouse model of visceral pain.

## Materials and methods

### Animals

Male Balb/C mice (Institute of Occupational Medicine, Lodz, Poland), weighing from 22 to 26 g, were used. Animals were maintained at a constant temperature (22–23 °C) under a 12-h light/dark cycle. Mice were housed in sawdust-lined plastic transparent cages with a free access to laboratory chow and tap water. All of the experiments were performed in accordance with respective national guidelines and animal use was approved by the Local Ethical Committee (#39/2016, #39-NZP/2018). In the in vivo experiments, each group consisted of 6–8 animals.

### Gastrointestinal motility

#### Contractility of isolated smooth muscle strips

Organ bath studies were performed as described previously (Zielińska et al. 2015). Briefly, full-thickness fragments (0.5 cm) of the colon were isolated and kept in Krebs solution (NaCl 115 mM, KCl 8.0 mM, KH_2_PO_4_ 2.0 mM, NaHCO_3_ 25 mM, MgCl_2_ 2.4 mM, CaCl_2_ 1.3 mM, and glucose 10 mM). One end of each intestinal fragment was attached to the bottom of the organ bath, and the other end to a FT03 isometric force displacement transducer (Grass Technologies, West Warwick, RI, USA) with a silk thread. The colon segments were placed longitudinally in individual organ between two platinum electrodes. The changes in tension were amplified by a P11T amplifier (Grass Technologies, West Warwick, RI, USA) and recorded on a personal computer using the POLYVIEW software (Polybytes Inc., Cedar Rapids, IA, USA). Electrical field stimulation (EFS; 8 Hz; 60 V; pulse duration 0.5 ms; train duration 10 s) was applied by a S88X stimulator (Grass Technologies, Warwick, RI, USA), and delivered through electrodes placed around the tissue.

Whole tissue intestinal segments (two intestinal preparations per one mice) were exposed to palvanil at increasing concentrations (10^−10^ to 10^−6^ M), added cumulatively into the organ bath (8 min for each concentration). At the beginning, the mean amplitude of three twitch contractions was measured and treated as an internal control. The changes in smooth muscle contractions were reported as the percentage of the internal control. In control experiments, the effect of the vehicle (dimethyl sulfoxide; DMSO) was assessed.

To assess the involvement of TRPV1 receptors, SB366791 (selective antagonist, 10^−6^ M) was added 10 min prior to palvanil.

#### Epithelial ion transport

Epithelial ion transport was assessed as described earlier (Fichna et al. 2009). Palvanil (10^−6^ M) or vehicle (DMSO) was added to the Ussing chamber after the establishment of baseline *I*_sc_ (15–30 min). Colonic samples were challenged with either forskolin (cAMP-dependent secretagogue activator, 10^−6^ M) or veratridine (voltage-dependent Na^+^ channel activator, 3 × 10^−5^ M) 10 min after exposition to palvanil (compound was added to the basolateral side, with concomitant dilution to a final concentration of 10^−6^ M). Open potential difference values were measured before and after the addition of palvanil/vehicle in order to calculate the conductance of tissue mS/cm^2^.

#### Whole gastrointestinal transit time

According to the protocol (Zielińska et al. 2017), whole GI transit time (WGT) was described as time between *i.g.* administration of blue marker (0.15 ml of liquid consists of 5% Evans blue and 5% gum Arabic) and first colored bolus excretion. To assess the influence of palvanil on WGT, palvanil (0.1, 0.25, or 1 mg/kg) or vehicle was injected intraperitoneally (*i.p.*) 15 min or 60 min before intragastric (*i.g.*) administration of colored dye.

#### Colonic expulsion test

The distal colonic expulsion test was performed in mice, fasted for 12 h according to the protocol described earlier (Zielińska et al. 2015). In our experiment, palvanil (0.1, 0.25, or 1 mg/kg) or vehicle was administered *i.p.* 15, 60, or 90 min before the insertion of prewarmed (37 °C) glass bead insertion into the colon (2.5 cm depth).

### Behavioral pain responses

Mustard oil (MO, allyl isothiocyanate)-induced pain test was performed as described earlier (Sobczak et al. 2014). Palvanil (0.1, 0.25, or 1 mg/kg) or vehicle was injected *i.p.* 15 or 60 min before MO administration. MO administration was followed by 5 min of recovery period. Then, spontaneous pain-related behaviors, such as licking of the abdomen, squashing of the lower abdomen against the floor, stretching the abdomen, and abdominal retractions, were counted for 20 min. The observation was performed by blinded experimenter.

### Drugs

All reagents, unless otherwise stated, were purchased from Sigma-Aldrich (Poznan, Poland). Palvanil was synthesized as previously described (Luongo et al. 2012; De Petrocellis et al. 2011).

In the ex vivo experiments, drugs were dissolved in dimethyl sulfoxide (DMSO). In the in vivo tests, drugs were dissolved in 5% DMSO in 0.9% NaCl.

### Statistics

Statistical analysis was performed using Prism 5.0 (GraphPad Software Inc., La Jolla, CA, USA). The data are expressed as a percentage of control values. In the ex vivo experiments, *n* indicates the number of individual tissue samples from ≥ 3 different animals. While in the in vivo studies, *n* stands for the number of animals. Student’s t-test was used to compare single treatment means with control means. Analysis of one way variance (ANOVA) followed by Newman–Keuls post hoc test was used for analysis of multiple treatment means. *P* values < 0.05 were considered statistically significant.

## Results and discussion

In our study, we focused on TRPV1 desensitization as a promising approach in the therapy of IBS. Palvanil, which was used in our study, is able to induce rapid desensitization of TRPV1 and is therefore devoid of pungency (in contrast to the parent compound capsaicin) (De Petrocellis et al. 2011). It was observed that palvanil caused TRPV1 desensitization nearly 5 times more potently than capsaicin; its maximal desensitizing effect was reached after 50 min of preincubation with palvanil, while capsaicin entailed a weaker effect after 5 h exposure. Moreover, it has been reported that 10 μl installation of palvanil (10 and 30 μg/ml) into the eye did not evoke eye-wiping movements in mice, whereas capsaicin (10 μg/ml) induced 12 ± 0.9 wiping reflexes within 30 s (De Petrocellis et al. 2011).

**Fig. 1:**
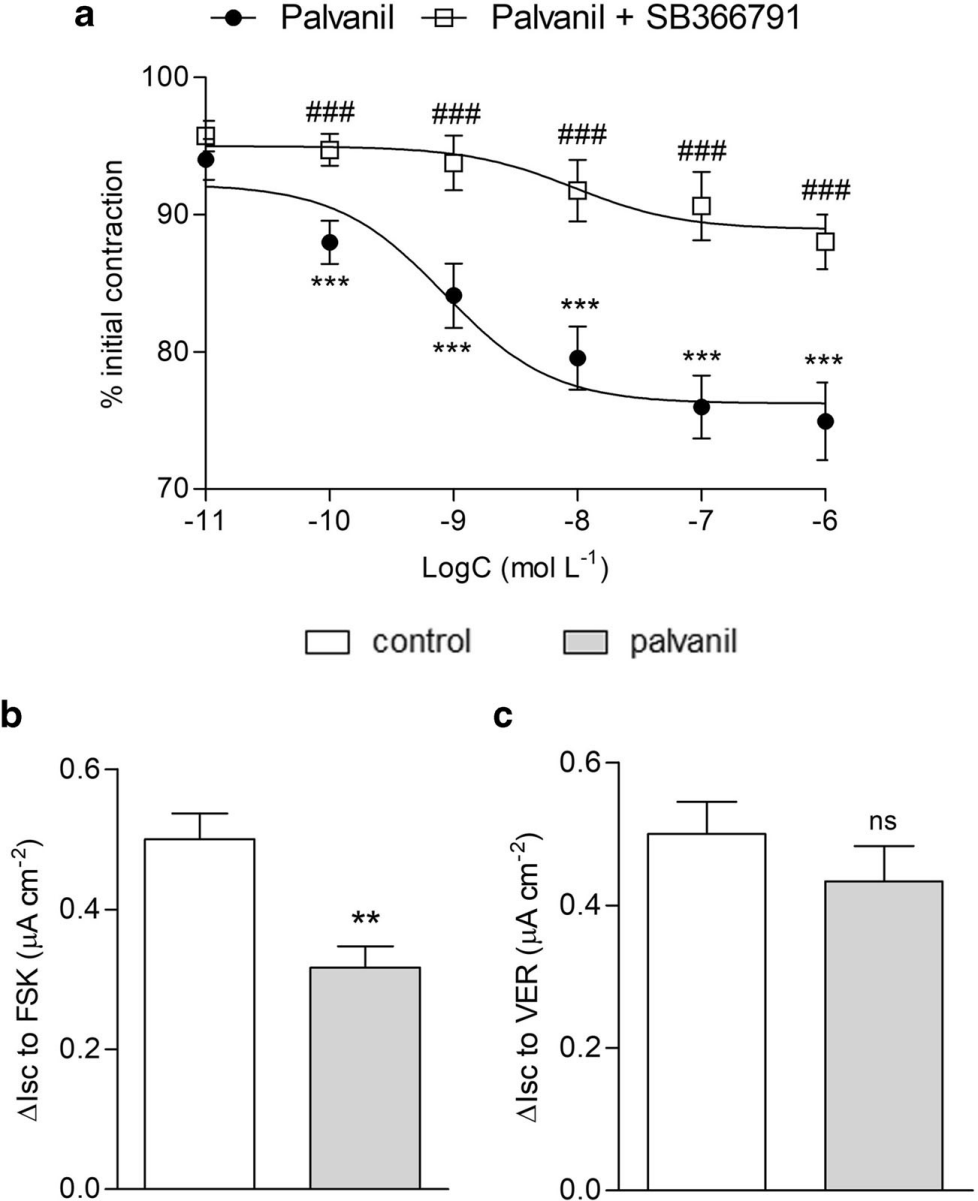
Panel A shows the inhibitory effect of palvanil (10^−10^–10^−6^ M) on EFS (8 Hz) stimulated longitudinal smooth muscle contractions in the mouse colon. The action of palvanil was reversed by the TRPV1 antagonist SB366791 (10^−6^ M). Data represent mean ± SEM for *n* = 6–8. ***P < 0.001 compared with control, ###*P* < 0.001 when compared with palvanil alone. Panel B and C present the influence of palvanil (10^−6^ M) on colonic secretion stimulated with forskolin (FSK, panel B) or veratridine (VER, panel C). **P < 0.01 as compared with control; ns, not statistically significant

In our study, palvanil (10^−10^–10^−6^ M) decreased the amplitude of longitudinal smooth muscle contractions in the EFS-stimulated distal colon in a concentration-dependent manner. We confirmed that its influence on colonic motility was TRPV1-depedent, as this effect was reversed by selective antagonist, SB366791 (10^−6^ M) (Fig. 1a). Similar inhibitory effect was reported by Rahmati (2012) who found that capsaicin (10^−9^–10^−7^ M) abolished motility in the jejunum by increasing the intervals between migrating motor complexes in C57BL/6 mice. This action was observed in WT animals, while it was absent in TRPV1 ^−/−^ mice. In contrast, capsaicin (10^−6^–10^−4^ M) induced sustained tonic increment in pressure (sustained contractions of the circular muscle layer) in both WT and TRPV1 ^−/−^ mice (Rahmati 2012). According to Maggi et al. (1997), high concentration of capsaicin (10^−4^–10^−3^ M) enhanced the EFS-stimulated contractions in circular muscle in the colon of the guinea pig. In mice, instillation of capsaicin resulted in rapid and transient contractions (primary effect) followed by delayed sustained contractions (secondary effect) in the distal colon and rectum. However, in the transverse and proximal colon, only the primary effect was noted. It was suggested that the effect of capsaicin depends not only on TRPV1 channels per se but also on their location in the colon (Matsumoto et al. 2009).

TRPV1 receptors are present on the epithelial cells of the alimentary tract, but their role in secretion remains unclear. Therefore, we characterized the effect of palvanil on epithelial ion transport (Fig. 1b, c). We observed that palvanil (10^−6^ M) decreased ion transport in the colon stimulated with forskolin (activator of adenylate cyclase, which promoted Cl^−^ and H_2_O secretion) when compared with control. The inhibitory effect on the ion transport in tissue stimulated with forskolin has been previously reported for capsaicin by Bouyer et al. (2013). It was observed that capsaicin (4 × 10^−5^ M) decreased the *I*_sc_ in the mouse colon stimulated with adenylate cyclase activator. Notably, it was determined that this effect was a result of Na-K-Cl-cotransporter 1 internalization rather than a direct action by TRPV1 receptors. In the guinea pig and human colon that were exposed to the H_2_S, the ability of excessive chloride secretion in TRPV1-dependent manner was reported (Schicho et al. 2006). This prosecretory effect was diminished by TRPV1 desensitization or by the action of TRPV1 antagonist, capsazepine (Savidge et al. 2002). Therefore, we presume that the inhibition of forskolin-induced secretion, revealed in our study, may be the result of rapid desensitization of TRPV1 by palvanil. On the contrary, in experiments with veratridine (an activator of voltage-dependent Na^+^ channels that promoted enteric neuron depolarization inducing Cl^−^ secretion through the epithelium in the colon (Hyland and Cox 2005)), palvanil did not affect ion transport in comparison with control.

**Fig. 2:**
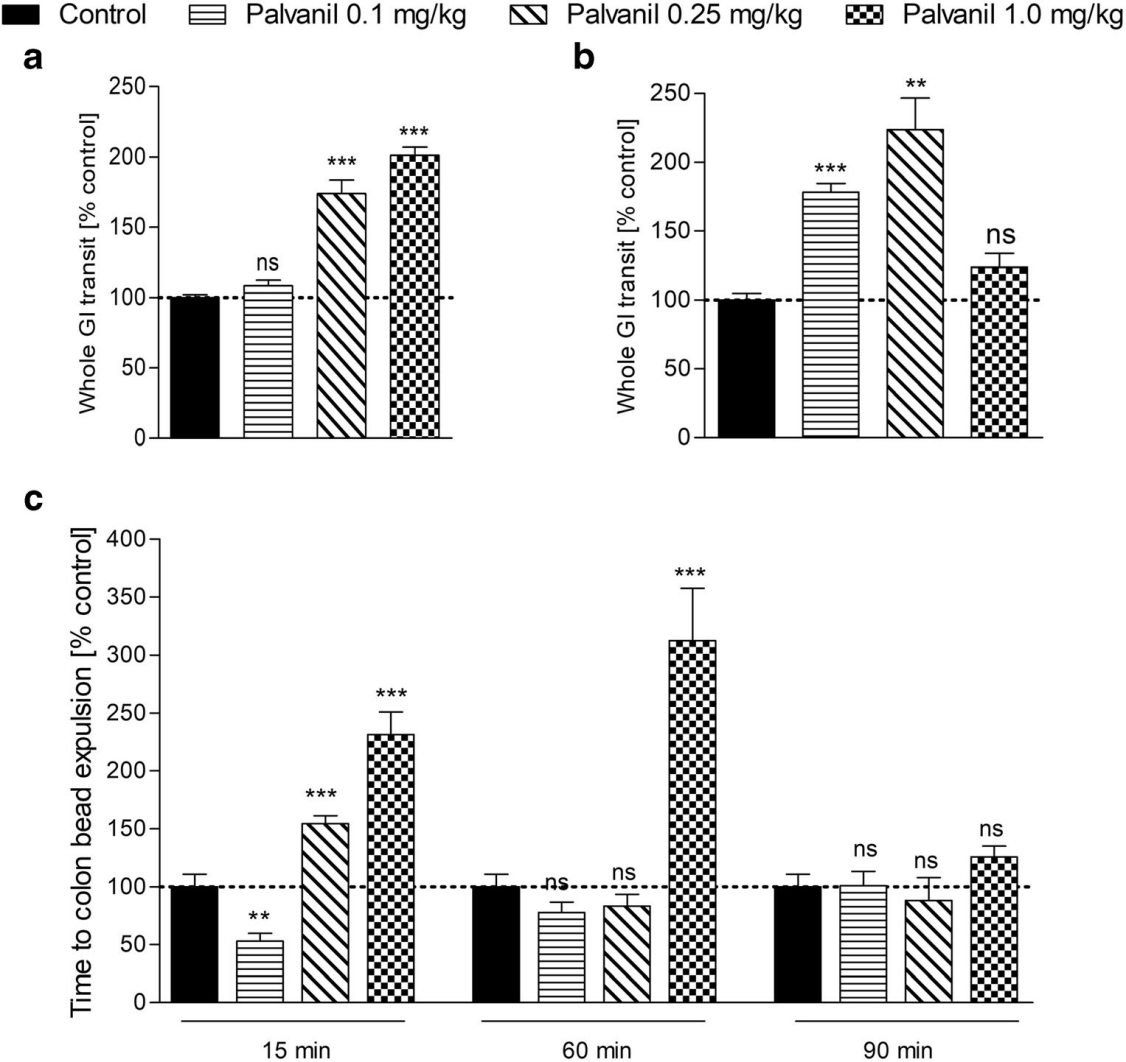
Palvanil (0.1–1 mg/kg, *i.p.*) elicited inhibitory effect on the whole GI transit time. Palvanil was administered 15 min (A) or 60 min (B) prior to colored marked gavage. Data are presented as % of the result obtained by mice from the vehicle treated group. The mean values of WGT time in control groups were 48.6 min (± 6.6) (panel A) and 66.6 min (± 10.7) (panel B). Each group contained 8 animals. **P < 0.01, ***P < 0.001 as compared with control; *ns* not statistically significant. (C) The effect of palvanil (0.1–1 mg/kg, *i.p.*) on the distal colon motility. Palvanil was administered 15, 60, or 90 min before the insertion of glass bead. Data are presented as % of the result obtained by mice from the vehicle treated group. The mean values of CB expulsion time in control group were 47.6 s (± 17.1), 80.0 s (± 24.1), and 160.7 s (± 28.5) for 15, 60, and 90 min, respectively. Each group contained 6–8 animals. **P < 0.01, ***P < 0.001 as compared with control; ns, not statistically significant

The systemic action of palvanil (0.1–1 mg/kg) was assessed in the whole GI transit test (Fig. 2a, b). Its efficacy was compared at two time points (15 or 60 min after its administration). Palvanil (0.25 and 1.0 mg/kg) administered 15 min prior to gavage of the colored marker significantly inhibited the GI transit. At the lowest dose of palvanil (0.1 mg/kg), there was no effect (Fig. 2a). When palvanil was administered 60 min prior to colored marker, the time of GI transit was markedly prolonged already at the dose of 0.1 mg/kg. At the highest tested dose (1 mg/kg), the transit time was only 23.8% longer than in control mice. However, this effect was not statistically significant (Fig. 2b). It was assessed that capsaicin (10 mg, administered intragastrically) possessed prokinetic effect in the upper GI tract (from the stomach up to the proximal jejunum) and in the colon but not in the distal jejunum and ileum in dogs (Shibata et al. 1999). The lack of effect of palvanil (1 mg/kg) in experiments with 60 min period before drug administration and colored marker gavage in our study may result from prokinetic effect in the upper GI tract combined with inhibited motility of the colon (as assessed in colonic beads expulsion test, see below) (Shibata et al. 1999).

We found that palvanil affects colonic motility in vivo: after *i.p.* administration, its action was the most prominent during the first 60 min. Palvanil at the dose of 0.1 mg/kg, 15 min following the injection, accelerated colonic motility. The opposite action was observed at higher doses (0.25 and 1 mg/kg, *i.p.*). In the experiments with longer time intervals, the action of palvanil was gradually abolished: 60 min after the injection, the inhibition of colonic motility was observed only for palvanil at the highest dose tested (1 mg/kg, *i.p.*) (Fig. 2c). The differences observed between doses could be comparable with the effects evoked by capsaicin in the mouse jejunum in the study of Rahmati (2012), mentioned above, or to the study in the distal colon, in which capsaicin at first induced rapid contractions (increase of intestinal motility) and then sustained contractions (prolongation of GI transit time). Hayashi et al. (2010) assessed how *i.c.* administration of capsaicin affects ileocolonic motility. It was observed that capsaicin (5 and 10 mg) induced defecation within 5–10 min following the enema. Both capsaicin at lower doses (1 or 2 mg) and placebo had no impact on motility or defecation. We observed similar trend in colonic bead expulsion test after intracolonic administration of palvanil: at the highest dose tested (1 mg/kg, *i.c.*), palvanil accelerated the expulsion of glass bead from the colon, while there was no result in experiments with lower dose of palvanil (0.1 and 0.25 mg/kg) (data not shown).

**Fig. 3:**
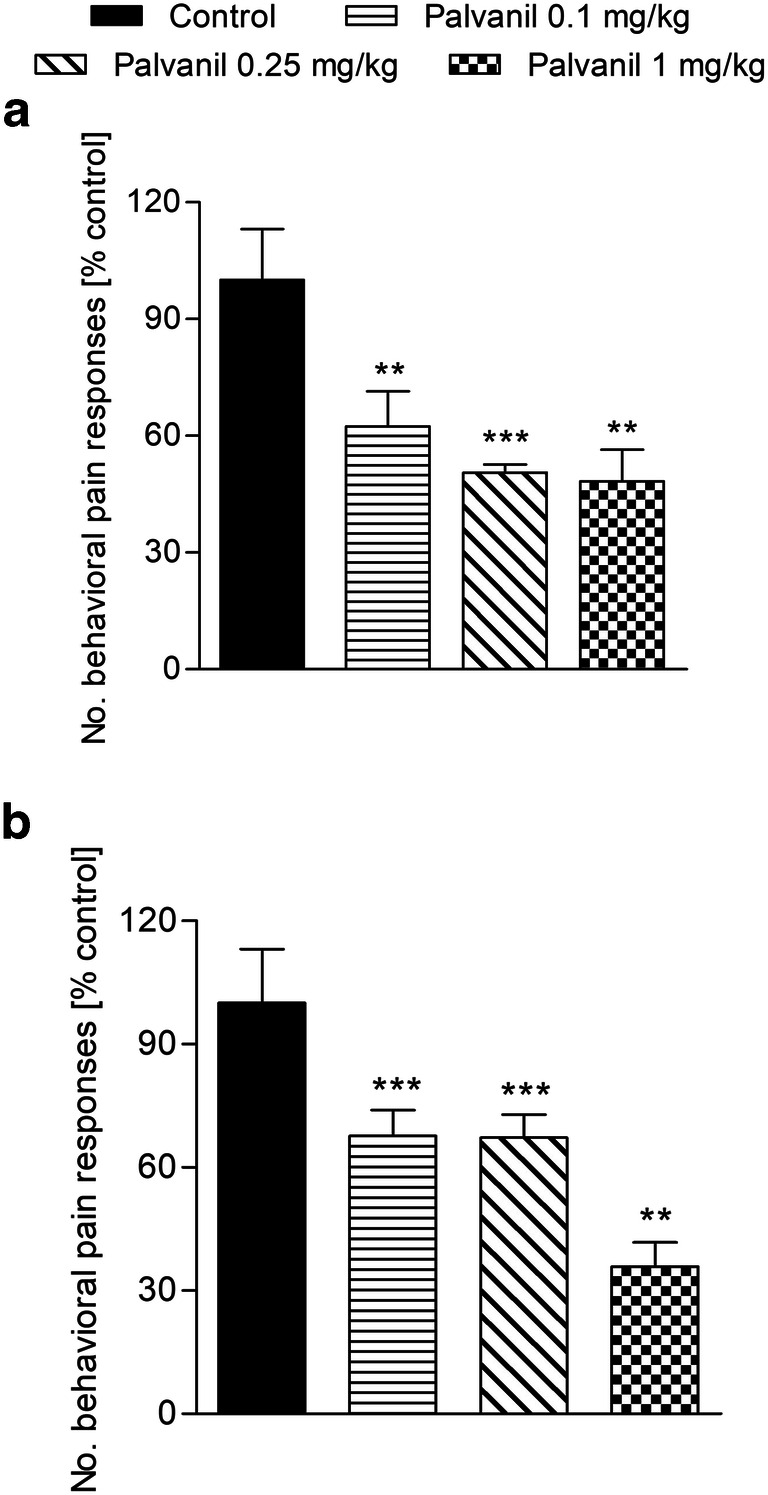
This figure presents the antinociceptive of palvanil (0.1–1 mg/kg) injected either *i.p.* 15 min (panel A) or 60 min (panel B) before the MO mustard oil installation. Data are presented as % of the result obtained by mice from the vehicle treated group. The mean numbers of pain-related behaviors in control groups were 44.3 (± 6.6) (panel A) and 43.8 (± 11.7) (panel B). Each group contained 6–8 animals. **P < 0.01, ***P < 0.001 as compared with control; ns, not statistically significant

To assess whether palvanil could possess a beneficial effect in alleviation of IBS-related symptoms, we characterized its action on visceral pain induced by MO *i.c.* instillation (Fig. 3). Experiments on antinociceptive action of palvanil varied by the drug dose and also the period between drug and MO administration (15 and 60 min). Mustard oil, despite being model agonist of TRPA1 receptors, also activates TRPV1 receptors: it was reported that TRPV1 ^−/−^ mice were insensitive to its irritant effect (Kistner et al. 2016). In our study, palvanil significantly reduced the number of behavioral pain responses up to 40% at all doses tested. Noteworthy, the antinociceptive action of palvanil at the dose of 1.0 mg/kg *i.p.* increased within the time of the experiment: palvanil administered 60 min prior to the test induced a more pronounced antinociceptive effect in comparison with experiments with a 15-min interval. Antinociceptive effect of palvanil in the distal colon is a result of TRPV1 receptors desensitization, which are subsequently irresponsive to noxious stimulation by MO. Similar relationship was reported for MO and capsaicin (Penuelas et al. 2007): in the distal colon of mice, the action MO on intestinal acetylcholine-mediated contractions was abolished in tissue incubated previously with capsaicin. The same effect was observed in opposite experiment: the amplitude of capsaicin-mediated contractions was lowered in tissue pretreated with MO. In our study, palvanil already 15 min following its administration desensitized the majority of receptors involved in nociception and thus, the irritant effect of MO was blocked.

Taking into account the physiological role of TRPV1 signaling, a safe therapeutic for IBS should attenuate the excessive contribution of TRPV1 (reported previously for IBS) and simultaneously preserve the physiological activity of these receptors. Therefore, desensitization appears to be an interesting alternative to the current therapeutic options. Palvanil, being devoid of pungent activity and able to counteract the excessive activation of TRPV1 in the course of GI diseases with visceral hypersensitivity (such as IBS), may emerge as superior to capsaicin in clinics. Moreover, palvanil, in contrast to its parent compound (capsaicin), modulates GI peristalsis, and therefore could improve an even broader spectrum of IBS-related symptoms. However, further studies on potential side effects and bioavailability are warranted.

### Contribution

MZ, JF, VDM, and MS designed the research study.

AS, MZ, JW, and AW performed the research.

AS, MZ, and JF analyzed the data.

AS, MZ, and JF wrote the paper.

All authors approved the final version of manuscript.
